# Port Wine

**Published:** 1861-01

**Authors:** 


### Port Wine.

This cerebral stimulant, tonic and astringent is one of the best of the wines to support the vital energies in adynamic diseases, particularly when debility of the digestive apparatus is a prominent difficulty. In Typhoid fever, a disease dependent on prostrating influences upon the great centres of vital or nervous power, leading to relaxed, passively ingorged, and indolently ulcerated conditions of the alimentary canal, as one of the necessary stimulants to the brain, it has properties peculiarly useful in giving tone to the viscera thus implicated. The most serious difficulty, however, in the use of most wines is, that we rarely get them pure, and often only in name. This is particularly true of port wine; yet while this is true, we have a compensation, in port at least, in the adaptation of the adulterating ingredients, such as logwood, &c., to the relaxed and ulcerated conditions referred to. And even in a spurious article, where no wine at all is found, but where common distilled spirits is added to the bogus compound, we may have the two important properties, astringency and cerebral stimulation. Notwithstanding we may often find the desired action in spurious wines, the state of things in this particular is to be deplored, and it is only by directing capital to wine growing in Georgia and other States where wine can be profitably raised, that the finer kinds of wine can be obtained pure.

The manner of manufacturing port wine, in the only country in which it is made is not exactly that which the most fastidious would prefer, yet we say, of every thing which we use let us have the pure article. Viewing this subject from a moral stand-point, we hesitate not to say, that so far from encouraging inebriety, the production of an abundance of pure wine in our midst would have a direct tendency to lessen the amount of bar-room drinking, and in that proportion, arrest the most dangerous habit of our youths. From the American Druggists' Circular we copy the following article on "Port Wine:"

PORT WINE.

"Port wine is from the province of Tras as Montes, (behind the mountains) on the north bank of the river Douro.—The scenery of this wine country is far from picturesque. The landscape simply consist of a series of high hills, covered with vines from base to summit, everywhere treeless, except for some elder clumps and a few olives here and there; but olive-trees are of sad countenance, subtantial friends of man, who do not offer him eye-service.

"The ground on the hills is a loose granite, with a very thin covering of soil, and it is cut into gigantic flights of steps, on which the vines are planted. These grow in bushes three or four feet high, about a yard part.

"The first care of the wine farmer, when his harvest-time approaches, is to engage men and women enough for the vintage work. The laborers engaged are almost savages, wild in their tempers, dirty in their persons and each male of them, man or boy, goes armed, after the custom of the province, with an ugly gun slung to his back. The day's food of these poor people is a little matter. They will think themselves very well off if they can get a couple of dried sardines for dinner, as a relish to their bit of Indian corn bread. The duty of the women in the vineyard is to put the bunches into large basket which the men carry upon their shoulders to the press. There is a great deal of singing on the ground, and all seem to work very contentedly, in spite of the great heat. When darkness ends the labor of the day, the laborers all meet outside the farm house, a guitar is produced, and dancing is kept up for some hours.

"When all the grapes are in the wine-press, the first thing to be done is to drag them well over with wooden rakes, to separate some of the stalks. Then all the men tuck up their trousers and jump in. At my friend's farm, a tub of water was ostentatiously set by the side of the press. I suspect, however, that this was a concession to the prejudice of visitors, for it did not go to the extent of actual ablution. Nobody used the tub of water, all seemed to have a supreme comtempt for cleanliness. The scene inside the press is very animated. Twenty or thirty brown-faced and black-bearded tatterdemalions, up to their knees in the purple juice, smoke, sing, quarrel, dance, scream, half mad with excitement, for to them this is the crowning event of the year. Every now and then a cry is raised for brandy, which the farmer furnishes. It is the pure white spirits as it has run from the still, and very strong. As it begins to take effect, the singing becomes louder, and the dancing, which within the press is the desired word, fast and furious. A general fight often ensues, in which the long guns sometimes play their part. When all the juice is trodden from the grapes, a plug is drawn.—The must runs through into a smaller tank, whence it is carried in buckets to the tuns, containing four or five pipes each, there to ferment.

"The wine-press is then half filled with water, the husks are again trodden, and finally squeezed under a press of wood. The liquor thus obtained ferments into what is called agua pe, a liquor that will be drunk by the laborers when they come, a month later, to prune the vines.

"When the fermentation of the wine in the tuns is complete the result would not suit English palates; being thin, and tart, and rough. It has, therefore, to be sweetened and fortified. For sweetening, geropiga is used. This is made by adding brandy to a part of the fresh must, which is thus prevented from fermenting, and retains, therefore, the sugar of the grape. Brandy is used to strengthen the wine. Often there is a deficiency of color, and this defect is cured with dry elderberries, tied in a sack, put into a tub about half full of wine. Into the tub a man gets, and by treading on the sack, soon draws the color from the berries, and the darkened liquor is added to the wine. This practice is common all over the wine country, and favorable spots are chosen for plantations of elder-bushes, solely to supply the demand for berries.

Port wine having being thus made, is racked out of the tuns into pipes, carried down by ox-carts to the river Douro, fastened to a barge, and floated to Oporto. There it is stored away till the time comes for shipping it to England; whither by far the greater part of the wine is exported. Only the superior class of wine is allowed to be sent abroad. Examiners appointed by the Board of Trade go round to taste and put their mark on those pipes they approve. Without their mark no cask can pass the custom house.

Of late years the yield of wine has been greatly diminished by the vine disease which first attacks the immature grapes in the form of a white powder, easily rubbed off. As the disease proceeds the powder changes to a fur, the grape turns black, and at last bursts, throwing out the seeds. The grape cluster then withers completely away; while the whole plant gives out a musty smell, very like that of toad-stools. The best known remedy is sulphur, sprinkled over the bunches with a pair of bellows, and for this purpose very large quantities of sulphur have been imported into Portugal. The failure of the wine crop is the most disastrous event that can happen to that country, for the wine farmers depend for life upon their grapes, the soil being too poor to produce any other crop.—*All the Year Round.*

